# Accounting for consumers’ preferences in the analysis of dietary recommendations

**DOI:** 10.1038/s41430-018-0317-5

**Published:** 2018-09-25

**Authors:** Linda Cobiac, Xavier Irz, Pascal Leroy, Vincent Réquillart, Peter Scarborough, Louis-Georges Soler

**Affiliations:** 1 Melbourne School of Population and Global Health, University of Melbourne, Melbourne, VIC Australia; 2 Natural Resources Institute Finland (Luke), Bioeconomy and Environment Research Unit, Helsinki, Finland; 3 Institut National de la Recherche Agronomique, INRA-ALISS, Paris, France; 4 Toulouse School of Economics, INRA, University of Toulouse Capitole, Toulouse, France; 5 Nuffield Department of Population Health, University of Oxford, Oxford, UK

**Keywords:** Cardiovascular diseases, Risk factors

## Abstract

**Background/Objectives:**

The goal of this article is to present and demonstrate the applicability of an original method to assess the economic and health impacts of compliance with food-based recommendations. The method takes account of consumers’ preferences and the associated adoption cost in the assessment of various recommendations.

**Subjects/Methods:**

We combine an economic model of diet choice with an epidemiological model to compute the health impacts of dietary changes. To demonstrate the use of the method, we analyse the impacts of a 5% variation in the consumption of seven food groups taken separately: a 5% increase in consumption of fruits and vegetables (F&V) and milk products; and a 5% decrease in consumption of red meat, all meats, salty/sweet products, ready meals and butter/cream/cheese.

**Results:**

A recommendation, when adopted by consumers, generates important changes in the whole diet due to substitutions and complementarities among foods. All simulated recommendations have a positive impact on health. The F&V recommendation has the largest impact on the number of DALYs averted, but the highest adoption cost for consumers, especially for low-income consumers. Alone, the change in energy intake explains from 71% to 98% of the DALYs averted induced by a recommendation.

**Conclusions:**

Small increases in recommended foods have the potential of generating relatively significant health gains. Preference-driven substitutions among foods have a major effect on simulated health outcomes and should be included in the assessment of dietary recommendations, together with the adoption cost borne by consumers.

## Introduction

The adoption of healthier diets is not easy for consumers [1, 2], as health is only one of many dimensions taken into account by consumers in the process of choosing foods [3]. Thus, the low rate of adoption of nutritional recommendations might be explained by economic factors (prices, limited income) [4, 5], but it is also caused by the ‘cost’ that compliance imposes on consumers in terms of modifying food habits. Indeed, even for health-aware consumers, the adoption of nutritional recommendations is difficult as it might lead to consume more of less-preferred foods or less of preferred foods [6]. Consumers may then incur, in the short term, an adoption cost—a ‘taste’ cost—which weakens their willingness to comply with recommendations. For this reason, beyond the research evaluating the health benefits of nutritional guidelines, an important issue is to better identify the compatibility of dietary recommendations with consumers’ preferences, in order to prioritize recommendations that generate health benefits while inducing low adoption costs for consumers.

Moreover, the adoption of a dietary recommendation affects the entire diet through complex product substitutions between- and within-food categories. For instance, a rise in fruits and vegetables (F&V) consumption may lead consumers to modify their consumption of meat or milk products, either because these products are substitutes or because of an indirect effect due to an income constraint. A popular way to construct diets that meet nutritional guidelines is to use linear programming models that provide optimized diets resulting from compliance with nutritional guidelines [7, 8]. However, an important weakness of these methods relates to the fact that substitutions between food groups are exogenously defined by the modeller rather than based on consumers’ preferences.

The goal of this article is to demonstrate the applicability of an original method to assess the potential health and well-being effects of compliance with dietary recommendations while taking into account consumers’ preferences and their effect on product substitutions within the diet. We use the economic theory of rational consumer choice, which allows the evaluation of the dietary changes compatible with revealed consumer preferences as well as the associated welfare cost of adoption of a recommendation. This model of diet choice is then combined with an epidemiological model in order to compute health impacts.

## Methods and data

### The economic model of diet choice

First, we use an economic model to predict the dietary adjustments that a rational consumer would make to satisfy a given recommendation [9]. Our approach strongly differs from that developed by nutritionists, who typically use optimization techniques to infer dietary changes [10–12]. Nutritionists assume that consumers, when complying with a set of constraints, seek to modify their diet as little as possible. The empirical implementation relies on the definition of a distance function between observed diet and modelled diet, followed by the minimization of that distance. For example, the distance function may be defined as the sum of the absolute values of the relative deviations between observed consumption and modelled consumption of each food product and each food group. Although this represents an attempt to integrate an element of consumer behaviour into the analysis, the definition of the distance function is arbitrary and neither based on a theory of consumer choice nor empirical data. Moreover, researchers generally add 'palatability' constraints, whose role is to limit substitutions deemed unrealistic. However, there is no strong justification for the addition of those constraints, which are arbitrarily set by the modeller.

In line with microeconomic theory, consumers are assumed to choose the goods (including foods) that they consume and their quantities so as to maximize their well-being, or utility, subject to a budget constraint reflecting prices and available income. Combining the theoretical solution to this ‘nutritionally unconstrained problem’ with data on actual consumption permits the estimation of price elasticities of demand for foods, which characterize consumers’ preferences. For instance, a low price elasticity of demand for a specific food group means that, even in the case of strong price increases, consumption does not change: this might be explained by taste preferences or cultural attachment to the product. Price elasticities provide information on the reaction of consumers facing a change in the relative prices of goods and are often used to compute the effects of fiscal policies (e.g., effects of fat and sugar taxes) [13, 14]. In some articles, consumer preferences are translated at the nutrient level, and nutrient elasticity estimates are used to calculate the health impact of various price policies [13, 15–17].

Within this framework, compliance with a dietary recommendation, such as that to consume five portions of F&V daily, is conceptualized as the addition of a constraint in the unconstrained programme, which gives the ‘nutritionally constrained problem’. The new constraint forces the consumer to adjust her choices, both in terms of which goods are consumed and their quantities, in order to satisfy that constraint. The two problems, constrained and unconstrained, are linked by ‘shadow prices’, which are defined as the set of prices that would have to prevail for the nutritionally unconstrained consumer to make the exact same choices as his/her nutritionally constrained equivalent. Thus, by definition, if shadow and market prices coincide, the consumer spontaneously satisfies the nutritional constraint. Using empirically estimated price elasticities, shadow prices can be calculated, from which follows the adjustment in consumption for each good so as to comply with the new constraint (i.e. a food-based recommendation).

The cost of adoption of a recommendation (also defined as a ‘taste cost’) is then measured by a ‘compensating variation’ (CV), defined as the additional income necessary to bring back the consumer to his initial level of well-being after he has adopted the recommendation. This CV is empirically estimable and quantifies the short-term loss of utility of the consumer who adopts a recommendation. Thus, the CV measures the difficulty of adopting a recommendation.

In this article, we used recent estimates of price elasticity of food demand available in France [13]. They are computed for four groups of consumers defined in terms of income levels (modest, lower average, upper average, well-off) by using food purchases recorded in a representative sample of about 5000 households by Kantar Worldpanel. Data on food consumption are taken from the French Food Survey INCA2^1^ providing the individual intakes in 2006 of French adult consumers (a detailed presentation of the different data sets is available elsewhere [9]).

### The PRIMEtime epidemiological model

Second, the epidemiological model PRIMEtime estimates the health effects of compliance with a recommendation by translating the dietary changes computed with the economic model into variations in the incidence of chronic diseases and mortality [18]. The PRIMEtime model is a proportional multi-state life table model [19], which simulates the life course of the current adult population of France and estimates incidence and mortality rates for diet and obesity-related diseases (cardiovascular diseases, cancers and kidney disease). Scenarios run through the PRIMEtime model in which the prevalence of dietary risk factors for disease in the population are altered and propagated into changes in disease incidence rates by the calculation of Population Impact Fractions [20] based on relative risks for disease taken from meta-analyses of epidemiological studies. Uncertainty analyses are based on a Monte Carlo analysis where random samples of the relative risks supporting the model are drawn from their underlying distributions. A full description of the PRIMEtime model including all of the underlying parameters of the model is available here [21].

The effect of dietary changes on population health is measured relative to a simulated baseline scenario, which describes a situation where current trends in incidence and case fatality from diseases continue into the future. Then, for each food-based recommendation the model is simulated reflecting the impact of the adoption of the recommendation by the whole population. The difference between the baseline and a simulated scenario is expressed through the number of averted disability-adjusted life years (DALYs). A DALY is a summary health outcome measure capturing both morbidity and mortality effects [22].

The PRIMEtime model is parametrized by using French data concerning the age structure of the population, the incidence of chronic diseases and mortality.^2^

### Adoption cost versus health benefit tradeoffs

In general, the cost effectiveness of a public health measure is defined as the ratio of its total cost and associated health effect. Measures are then judged to represent ‘good value for money’ if that ratio falls under a threshold [23, 24]. In most cases, costs refer to policy and healthcare costs but, in our case, consumers changing their diets also bear a utility cost, i.e. an adoption cost. Thus, we compute the consumer cost per DALY and discuss whether its amount is likely to modify the cost effectiveness of the simulated recommendation. To do so, we compute the ratio of the CV and the number of DALYs saved per year, which is estimated as a tenth of the number of DALYs averted over a ten-year period as calculated by PRIMEtime.

### Simulated food-based recommendations

In line with recent dietary guidelines updated by the French national agency (Anses)^3^, the economic model was used to simulate the dietary changes induced by a 5% variation in the consumption of seven food groups taken separately: a 5% rise in consumption of F&V and dairy products; a 5% reduction in consumption of red meat, all meat, salty/sweet products, ready meals and butter/cream/cheese.

## Results

To illustrate the dietary changes induced by the adoption of a recommendation and the mechanisms that explain the changes, we first present the impact of a 5% increase in F&V consumption. Table 1 displays the intakes (g/day) of the food categories considered in the economic model, the shadow prices corresponding to the F&V recommendation, and the percentage variations in consumption of each food category induced by the rise in F&V consumption, including the F&V contained in ready meals.

**Table 1:** Initial intakes, shadow prices and variation in the whole diet induced by a 5% increase in F&V consumption (men, well-off consumers)

	Baseline daily intakes (g/day)	Shadow prices (variation as compared to current prices)	Variation in daily intakes induced by the the adoption of the F&V recommendation (5% increase)
Red meat	41	0%	−5.4%
Other meats	57	0%	3.5%
Cooked meats	46	0%	−1.9%
Fish & seafood	37	0%	2.4%
Eggs	17	0%	−4.5%
Grains	238	−0.2%	−3.9%
Potatoes	59	0%	−17.0%
Fruits—fresh	186	−19.8%	4.1%
Fruits—processed	13	−13.9%	13.2%
F&V juices	50	−9.4%	1.9%
Vegetables—fresh	189	−19.3%	7.1%
Vegetables—processed	29	−12.9%	8.7%
Fruits—dry	5	−3.7%	−2.3%
Milk products	157	0%	−2.6%
Cheeses, butters, fresh creams	60	0%	−2.0%
Ready meals	127	−1.9%	−6.2%
Oil, margarine, condiments	24	0%	7.4%
Salt-fat products	24	0%	−12.5%
Sugar-fat products	130	−0.8%	1.5%

As shown in Table 1, the shadow prices, which are the prices that would have to prevail to lead spontaneously consumers to increase their F&V consumption by 5%, are lower than current prices for all food groups that contain some F&V. The increase in F&V consumption generates a change in the whole diet. First, the 5% increase in F&V consumption is achieved thanks to a rather large increase in processed F&V, meaning that it is easier for consumers to increase consumption of processed F&V than fresh F&V. The decrease in potatoes and ready-meals consumption is easily understood as a substitution among plant-based foods. The decrease in consumption of milk products (fresh dairy products) is also understood as a substitution with fruits. Finally, there is a decrease in consumption of red and processed meats and an increase in consumption of other meats (poultry), which is likely caused by an income effect.

The adoption cost associated with the adoption of the recommendation is very different for the different consumer groups (Table 2). Thus, the CV ranges from 0.2% of the food budget for the well-off group to 1.6% of the food budget for the modest group, suggesting that the adoption of this recommendation is more difficult for low-income consumers than high-income consumers.

**Table 2:** Aggregated consumer cost per income groups

F&V	Taste cost (million euros per year)	% food budget
Modest	310	1.63
Lower average	109	0.50
Upper average	23	0.11
Well-off	25	0.15
Total	466	

**Fig. 1:**
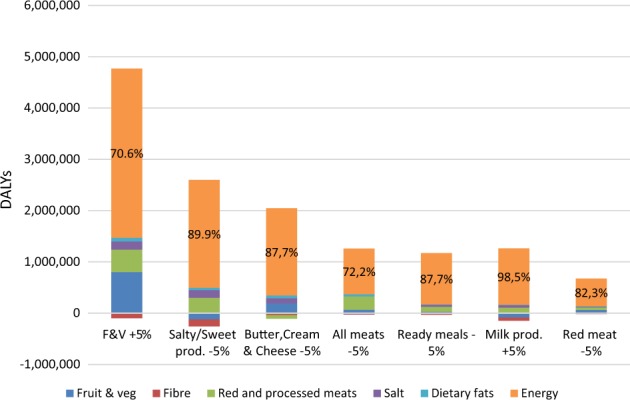
Impact of dietary risks factor, considered in the PrimeTime model, on net health gains induced by the adoption of food-based recommendations over the life cycle Note: The values reflect the change in total DALYs if the risk factor is eliminated from the analyses. A negative value indicates that the change in risk factor is leading to a loss of health. For example, in the case of the recommendation on salty/sweet products, the change in F&V consumption negatively impacts the health. This is because the adoption of the recommendation leads to a decrease in F&V consumption. The sum of DALYs across all risk factors does not equal the total DALYs from the modelling combining all risk factors because the potentially avertable disease burden diminishes with each additional risk factor. In the orange area, the % refers to the contribution of the reduction of energy intake to the net gain

If we now turn to the health impact of the seven tested recommendations, it appears that they all improve health as the number of DALYs averted is always positive (Fig. 1). The top three recommendations are the F&V recommendation, which has the largest health impact, recommendations on salty/sweet products, and butter/cream/cheese. Finally, the recommendation targeting consumption of red meat generates the smallest number of DALYs averted.

Alone, the change in energy intake explains from 71% (recommendation on F&V) to 98% (recommendation on milk products) of the DALYs averted due to the adoption of a recommendation. The changes in intakes of F&V and red/processed meats have on average similar relative health impacts but, in some cases, those impacts can be negative. This is when a recommendation leads to a decrease in intake of F&V (e.g. recommendation on salty/sweet products) or an increase in red and processed meats (e.g. the recommendation on butter, cream and cheese). The health impact of changes in intakes of salt and dietary fats is generally small but positive.

**Fig. 2:**
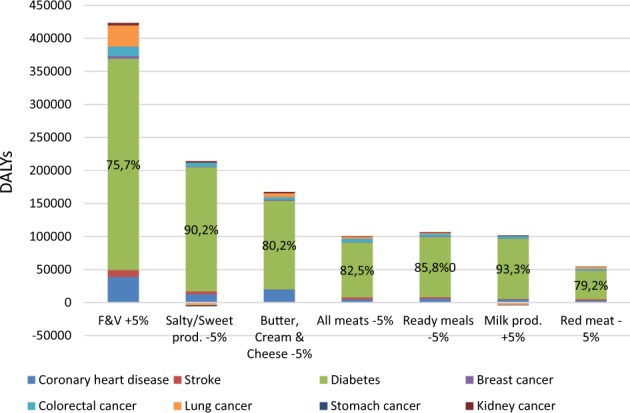
Incident cases averted in first ten years thanks to the adoption of food-based recommendations in the French population. Note: in the green area, the % refers to the share of incident cases averted due to reduction in diabetes

The adoption of the simulated recommendations reduce the number of incident cases of the different diseases considered in the analysis (coronary heart disease, stroke, diabetes and different types of cancers). However, the largest impact is on type 2 diabetes (Fig. 2). It is by far the disease which registers the largest number of incident cases averted (from 76% to 93%).

The adoption of a recommendation is costly for consumers as it means departing from their optimal (current) choices. However, depending on the recommendation, the cost of change, interpreted as a adoption cost, can be high or low (Table 3). For example, reducing intake of red meat by 5% generates little cost. Similarly, for several recommendations, adoption costs are lower than €15 m per year for the French adult population. On the other hand, the population-level adoption cost of adopting recommendations on all meats, salty/sweet products and butter/cream/cheese ranges between €75 m and €110 m per year. Finally, the recommendation on F&V intake is the most costly, with a total cost worth about €460 m annually.

**Table 3:** Number of DALYs averted, consumer cost and cost per DALY averted for the seven food-based recommendations

	F&V + 5%	Salty/sweet products −5%	Butter, cream & cheese −5%	All meats −5%	Ready meals −5%	Milk products + 5%	Red meat −5%
**DALYs averted in the first ten years (thousands)—mean and 95% incertainty interval**							
Men	146 (138–154)	82 (77–87)	59 (54–63)	41 (39–43)	39 (36–41)	39 (36–55)	20 (19–22)
Women	117 (110–123)	67 (62–71)	52 (48–56)	32 (30–34)	32 (30–34)	32 (30–34)	17 (16–18)
Total	263 (248–277)	149 (139–158)	111 (103–118)	73 (69–77)	71 (67–75)	71 (66–88)	37 (35–40)
Ranking of recommendations (DALYs)	1	2	3	4	5	5	7
Annual taste cost (millions of euros)	466	89	109	76	10	13	10
Ranking of recommendations (taste cost)	7	5	6	4	1	3	1
Taste cost/DALY (euros)	17720	5970	9820	10140	1410	1830	2700
Ranking of recommendations (taste cost/ DALY)	7	4	5	6	1	2	3

The adoption cost per DALY ranges from around €2000 (ready meals, milk products, red meat) to about €17,000 (F&V), meaning that it is less costly for consumers to avert one DALY by reducing consumption of ready meal and red meat, or by increasing consumption of milk products rather than by increasing consumption of F&V.

## Discussion

In this article, we proposed an original approach to assess the compatibility of dietary guidelines, regularly published by public health authorities [25, 26], with consumers’ preferences, and produce estimates of how different recommendations affect health outcomes as well as consumers’ welfare at the margin (i.e., for realistic, small changes in intakes). The empirical application to seven recommendations in France demonstrates the usefulness of the approach and supports several conclusions.

Substitutions among foods have a major effect on estimated health outcomes, which confirms the need to introduce realistic preferences and consider whole diets in the analysis of nutritional health policies. In particular, the results establish that a large share of the health gains are due to changes in dietary calories rather than improvements in diet quality, although that type of adjustments are usually ignored by assumption in studies of dietary recommendations through the imposition of iso-energy constraints [27]. The study also shows that a single food-based recommendation typically results in an improvement in diet quality in multiple dimensions. For example, an increase in consumption of F&V induces a reduction in consumption of red and processed meats. This result is in line with a recent ex-post evaluations of the UK 5-a-day campaign, which estimated that the increase in F&V consumption induced by the policy (about 5%) was associated with other significant dietary adjustments, and most noticeably a decrease in red meat consumption [28]. Substitutions matter a great deal because, in some cases, the adoption of a recommendation might also generate unintended adverse effects in some dimensions of the diet. Altogether, and in view of the complex adjustments simulated by our model on the basis of empirically estimated preferences, the common practice of imposing 'palatability constraints' in models of diet optimization does not appear fully satisfactory on both theoretical and empirical grounds.

The decrease in consumers’ short-term well-being that we measure as the adoption cost with recommendations can be large when measured at population level (e.g., almost half a billion euros annually for a 5% increase in F&V). In many cases, such large private costs are likely to exceed direct policy costs and should therefore be included in cost-effectiveness analyses although, to the best of our knowledge, that has never been done when assessing nutritional health interventions. The significance of the adoption costs also explains the non-adoption of recommendations, and provides a measure of the relative difficulty of compliance with different recommendations. In any case, the adoption cost per DALY is substantial and should be therefore integrated in the evaluation of the cost effectiveness of nutritional and dietary recommendations.

Overall, recommendations to increase consumption of F&V or decrease consumption of salty/sweet products and butter/cream/cheese have the largest health impact as measured by the number of DALYs averted, but they also generate the largest adoption cost for consumers. Conversely, other recommendations, such as those seeking to raise consumption of milk products or decrease consumption of ready meals, have lower health impacts but impose small adjustment costs on consumers.

Because the dietary adjustments to recommendations depend on consumers’ preferences, they are expected to vary within a population. Our method permits quantification of the relative difficulty of adoption of recommendations and could be used to estimate how this difficulty varies across types of consumers, which should help policymakers select appropriate nutritional health campaigns depending on the target group of interest. Another relevant application of the method would be to develop comparative analyses across countries.

We must also acknowledge some limitations. The method only allows for marginal dietary changes (here, a 5% variation in the consumption of different food groups), as it is based on elasticities estimated from observation on current consumption patterns. Thus, our approach should be seen as a complement to usual approaches based on linear programming to optimize diets [29]. While the latter group of methods can be used to define long-term dietary targets, our model is useful to identify incremental changes along a path of least resistance towards those targets. A second limitation is linked to uncertainties surrounding the price elasticities estimates, which we did not investigate in a sensitivity analysis. Another issue is related to the heterogeneity of consumers which is not taken into account in our approach as we use elasticities for four representative consumers. Heterogeneity might come from numerous characteristics of consumers, and most notably cultural and religious characteristics. Nevertheless, while recognizing that more remains to be done, we hope that our analysis makes a convincing case that preference-driven substitutions and adoption costs of dietary adjustments should be given due consideration in future analyses of dietary recommendations.

## Footnotes

1 See https://www.anses.fr/en/content/detailed-results-inca-2-study

2 The source of the data is the French Institute of Research on Human Health (INSERM) (https://www.inserm.fr/en)

3 See https://www.anses.fr/en/content/anses-updates-its-food-consumption-guidelines-french-population
